# Artificial intelligence applicated in gastric cancer: A bibliometric and visual analysis *via* CiteSpace

**DOI:** 10.3389/fonc.2022.1075974

**Published:** 2023-01-04

**Authors:** Guoyang Zhang, Jingjing Song, Zongfeng Feng, Wentao Zhao, Pan Huang, Li Liu, Yang Zhang, Xufeng Su, Yukang Wu, Yi Cao, Zhengrong Li, Zhigang Jie

**Affiliations:** ^1^ Department of General Surgery, The First Affiliated Hospital of Nanchang University, Nanchang, China; ^2^ Medical Innovation Center, The First Affiliated Hospital of Nanchang University, Nanchang, China; ^3^ Jiangxi Med College of Nanchang University, Nanchang, China; ^4^ The Third Clinical Department of China Medical University, Shenyang, China

**Keywords:** gastric cancer, bibliometric analysis, CiteSpace, research trends, Web of Science, artificial intelligence

## Abstract

**Objective:**

This study aimed to analyze and visualize the current research focus, research frontiers, evolutionary processes, and trends of artificial intelligence (AI) in the field of gastric cancer using a bibliometric analysis.

**Methods:**

The Web of Science Core Collection database was selected as the data source for this study to retrieve and obtain articles and reviews related to AI in gastric cancer. All the information extracted from the articles was imported to CiteSpace to conduct the bibliometric and knowledge map analysis, allowing us to clearly visualize the research hotspots and trends in this field.

**Results:**

A total of 183 articles published between 2017 and 2022 were included, contributed by 201 authors from 33 countries/regions. Among them, China (47.54%), Japan (21.86%), and the USA (13.11%) have made outstanding contributions in this field, accounting fsor 82.51% of the total publications. The primary research institutions were Wuhan University, Tokyo University, and Tada Tomohiro Inst Gastroenterol and Proctol. Tada (n = 12) and Hirasawa (n = 90) were ranked first in the top 10 authors and co-cited authors, respectively. Gastrointestinal Endoscopy (21 publications; IF 2022, 9.189; Q1) was the most published journal, while Gastric Cancer (133 citations; IF 2022, 8.171; Q1) was the most co-cited journal. Nevertheless, the cooperation between different countries and institutions should be further strengthened. The most common keywords were AI, gastric cancer, and convolutional neural network. The “deep-learning algorithm” started to burst in 2020 and continues till now, which indicated that this research topic has attracted continuous attention in recent years and would be the trend of research on AI application in GC.

**Conclusions:**

Research related to AI in gastric cancer is increasing exponentially. Current research hotspots focus on the application of AI in gastric cancer, represented by convolutional neural networks and deep learning, in diagnosis and differential diagnosis and staging. Considering the great potential and clinical application prospects, the related area of AI applications in gastric cancer will remain a research hotspot in the future.

## 1 Introduction

Gastric cancer (GC) is one of the most important members of the global cancer burden (1). According to the 2018 Global Cancer Statistics Report, the number of GC cases has exceeded 1,034,000, and it has become the fifth most common cancer (5.6%) except breast (11.7%), lung (11.4%), colorectal (10.0%), and prostate cancer (7.3%) (2, 3). Due to its frequently advanced stage at diagnosis, gastric cancer is with high mortality. Additionally, it was estimated as responsible for about 783,000 deaths worldwide in 2018 and was the third leading cause of cancer death worldwide (4).

Endoscopic ultrasound (EUS), MRI, computed tomography (CT), positron emission computed tomography (PET-CT), and other imaging examinations recommended by the guidelines for GC of the Chinese Society of Clinical Oncology (CSCO) in 2021 played an important role in the clinical diagnosis, therapeutic evaluation, and prognosis prediction of GC (5). The majority of early gastric cancer (EGC) can be cured by endoscopic resection, and the 5-year survival rate can reach more than 90%. In addition, comprehensive surgical treatment is the main treatment for advanced GC; the 5-year survival rate of advanced GC is <30%. It can be concluded that early detection, early diagnosis, and treatment of GC is the main strategy to reduce the mortality and improve the survival rate. Significantly, the rapid development of endoscopy has increased the detection rate, diagnosis, and treatment rate of EGC, and increased the incidence postoperative quality of life (6, 7). However, the professional knowledge and experience of the endoscopist and complex factors of the gastrointestinal (GI) tract determine the accuracy of detection (8). At the same time, with the era of individualized treatment of GC, the obvious deficiency of biological information such as morphology, size, and enhancement of the lesion of traditional imaging examination reflecting tumor heterogeneity is gradually exposed (9–11). Every area and industry, including healthcare, has been influenced by digital transformation as a result of the synchronous maturation of several significant digital innovations in information and communications technology that developed at an unprecedented rate this new century. Meanwhile, a remarkable ecosystem for new opportunities in healthcare and other industries has been created by digital innovations including the further consolidation of tele-health, the evolution of fifth generation wireless networks (5G), artificial intelligence (AI) approaches such as machine learning (ML) and deep learning (DL), and the Internet of Things (IoT), and digital security capabilities such as blockchain (12). The concept of AI was first proposed in 1956 and has developed rapidly in the past 10 years. The intersection of medical and engineering disciplines resulted in the development of AI-based radiomics and deep-learning technology, which can overcome the limitation of conventional imaging that depends on visual judgment, convert images into massive data features that can be mined, and objectively and quantitatively represent the heterogeneity and microenvironment within tumors. Therefore, AI technology has shown great advantages in the clinical diagnosis, therapy, and prognosis prediction of GC, which is a hotspot of research at present (13). Pathology, endoscopy, and computed tomography (CT) were mainly included in AI-assisted diagnosis, while prognosis researchers concentrated on recurrence, metastasis, and survival prediction (14).

In this study, scientometric analysis is mainly reflected in bibliometric and manual analysis. Bibliometric analysis is a new tool for exploring patterns and trends of a filed or subject at a high rate of speed using statistical methods and visualization (15). Moreover, CiteSpace, a Java-based application, uses metrology, co-authorship, co-citation, and co-occurrence analyses between countries, institutions, journals, authors, references, and keywords to quantitatively and qualitatively analyze and visualize the current research status and research frontiers in a certain field (16, 17). In the past few years, relatively many researchers and institutions have focused on the research of AI in the field of GC. Based on the core collection database of Web of Science, this study adopts CiteSpace knowledge graph software technology to understand and compare the basic situation, research hotspots, and development trends of AI in the field of GC from the perspective of visualization, so as to provide new ideas and clues for related research work.

## 2 Methods

### 2.1 Data source

With the availability of bibliometric indicators and more than 12,000 significant high-quality journals from nations throughout the world, Web of Science (WoS), one of the most comprehensive, systematic, and authoritative databases, is widely used for bibliometrics analysis and visualization of scientific literature (18). Significantly, the data collected from WoS could directly provide reference files that satisfy the specific format requirements set by bibliometric software CiteSpace. Otherwise, if data were downloaded from other databases, an additional procedure for file format conversion should be required (19). In addition, the accuracy, reliability, and representativeness of a certain dataset rely on the authority of the database, so the Science Citation Index Expanded (SCI-Expanded) of Web of Science Core Collection (WoSCC) database was chosen as the data source in this study. With allowing us to extract the relevant publications using an appropriate retrieval strategy, the majority of publications on AI applicated in GC are included in the WoS online database.

### 2.2 Retrieval strategies

**Figure 1:**
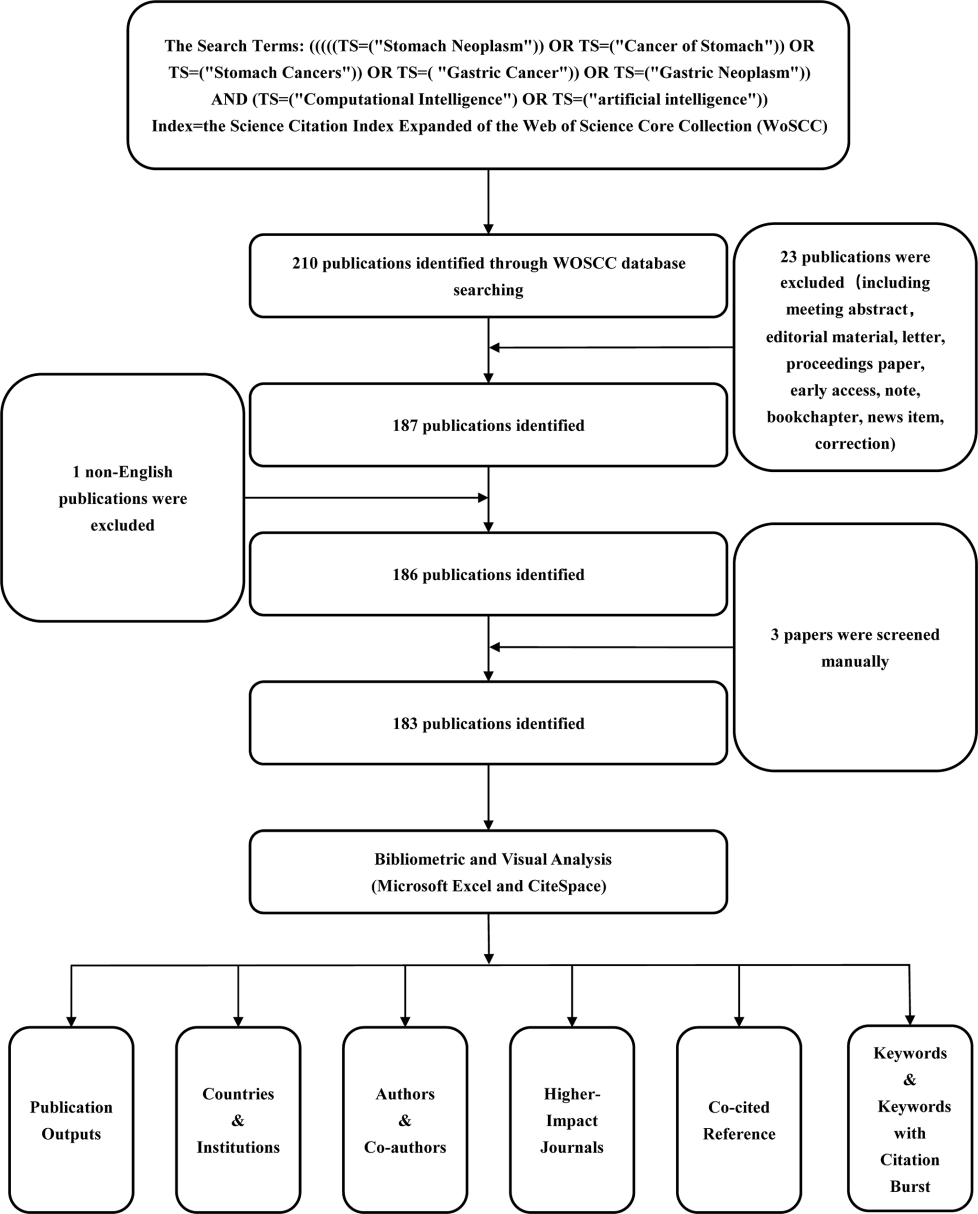
Flowchart of literature selection and the steps of bibliometric analysis included in this study.

A flow chart of literature selection included in this study is shown in **Figure 1**. Publications were retrieved from the Science Citation Index (SCI) Expanded of the WoSCC database from 2017 to 2022 and were downloaded within 1 day on 6 October 2022 in order to reduce bias due to daily updates of the database. The search terms were as follows: (((((TS=(“Stomach Neoplasm”)) OR TS=(“Cancer of Stomach”)) OR TS=(“Stomach Cancers”)) OR TS=(“Gastric Cancer”)) OR TS=(“Gastric Neoplasm”)) AND (TS=(“Computational Intelligence”) OR TS=(“artificial intelligence”)). In addition, to ensure the accuracy and objectivity of the analysis, only articles and reviews written in English were included, and other types of publications, such as meeting abstract and editorial material, were excluded. Finally, there remained a total of 183 publications related to this field, and then they were exported as “full records and references.” All records, including the titles, authors, abstracts, keywords, etc., were imported to CiteSpace to summarize and visualize scientific literature.

### 2.3 Analysis tool

The records retrieved and downloaded from the WoSCC were converted into plain text format for export, which was named download_ XXX.txt, including complete records and references. Finally, all valid data retrieved from the WoSCC was imported into Microsoft Excel and CiteSpace to perform a bibliometric and visual analysis.

CiteSpace, an interactive visualization analysis software developed by Professor Chen Chaomei of Drexel University using Java language based on scientometrics and data visualization, cannot only analyze the distribution of countries/regions, authors and co-cited authors, journals and co-cited journals, co-cited references, keyword cluster analysis, and timelines but also support structural and temporal analyses of a variety of networks derived from scientific literature, including collaboration networks, co-occurrence networks, co-citation networks, and networks of hybrid node types such as terms, authors, and countries (20–22). Additionally, burst detection is also one of the functions of CiteSpace, which can be used for detecting sharp increases of interest in a certain field (23). The results of the bibliometric analysis were displayed as knowledge network maps created by CiteSpace, and knowledge network maps is a growing field motivated by digital technology to easily understand the research hotspots and evolution process of different fields in the knowledge system and predict the development trend of various fields (24). As a consequence, we can concentrate on examining patterns and dynamic changes in scientific research publications and try to identify key points in a certain field to evaluate the current research hotspots and development tendency in a certain filed through the distribution and structure of scientific knowledge presented by knowledge map. In addition, the obtained data were exported into R Studio (Bibliometrix: An R-tool version 3.2.1) and VOSviewer (version 1.6.17) to draw scientific knowledge maps visually.

## 3 Results

### 3.1 Trends of publication outputs

**Figure 2:**
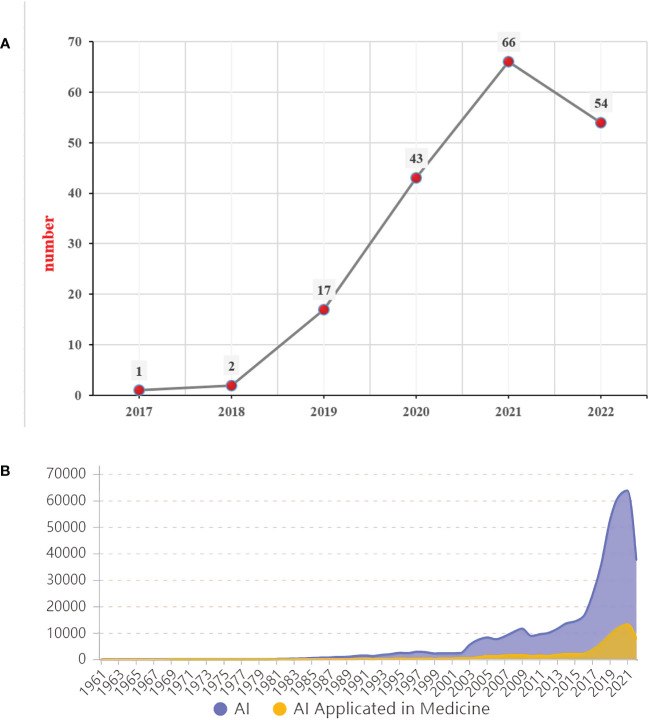
**(A)** Change trend of annual number of publications related to the research on AI applicated in gastric cancer, 2017–2022. **(B)** Change trend of number and the annual number of publications related to the research on AI and AI applicated in medicine, 1961–2022.

As shown in **Figure 2A**, the data of a total of 183 publications in this research field from 2017 to 2022 were retrieved and plotted *via* WoS database, and the earliest literature on the application of AI in the field of GC in the database was published in 2017, but the number of publications was only 1. The change trend in number and the annual number of publications related to the research on AI and AI applicated in medicine are displayed in **Figure 2B**, and we can observe that it has been a stage of rapid development of AI since the 1990s. Especially, the number of relevant publications has shown a rapid increase since 2016, and the annual growth rate of the number of publications from 2016 to 2017 was the highest, reaching 49.20%. At the same time, the application of AI in the field of medicine has also developed accordingly, and the number of publications has also increased year by year. Similarly, AI also shows great application prospects in transportation, education, finance, and other fields. Since 2017, research on AI in the field of GC has also received corresponding attention, and the number of relevant articles has been increasing annually. Particularly in 2021, the annual average growth rate reached 264.11%. However, the overall base of research on the application of AI in the field of GC was small, which indicated that there were still few relevant studies in this field.

### 3.2 Quantitative and cooperation analysis

#### 3.2.1 Bibliometric analysis of countries and institutions

In the cooperative network map created by CiteSpace software, the larger the size of the node that represents research items such country/region, institution, and author, the more frequently the item appears or is cited. In addition, a series of citation tree-rings across a number of time slices are used to describe each node. The lines between nodes represent a co-occurrence or a co-citation between these nodes, and the thicker the line, the closer is the connection between them (25). The distinction of different years is represented by different colors. Moreover, the outermost layer of the node is surrounded by a purple circle, and the size of this purple proportion represents the centrality of each item. In CiteSpace, the term “centrality” refers to intermediary centrality and is an indicator to assess the significance of each network node (26). CiteSpace uses this index to evaluate and reveal the importance of the literature in a certain field, and if node-intermediary centrality with purple is not <0.1, then such node will be applied a purple circle for emphasis.

**Figure 3:**
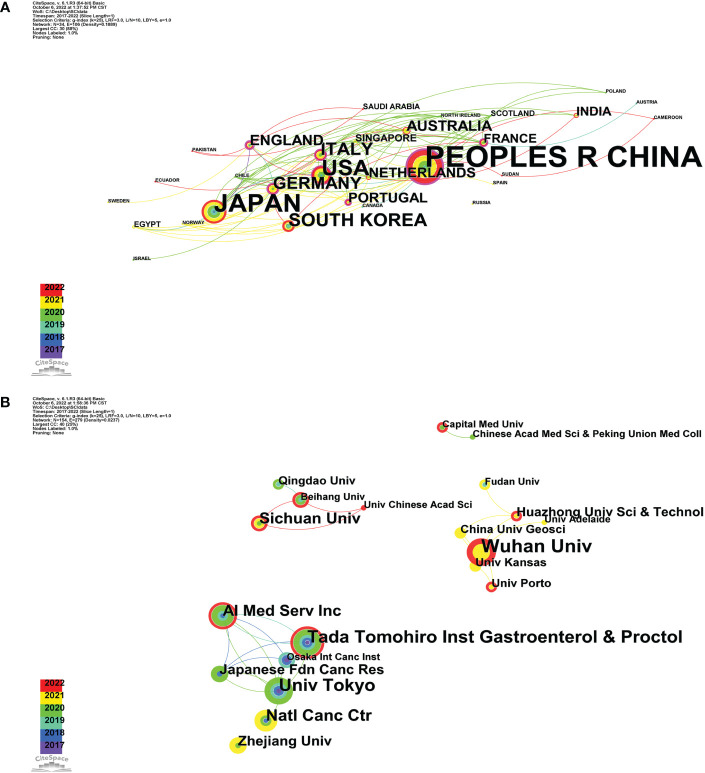
**(A)** Map of a subset of cooperative relations among countries, 2017–2022. **(B)** Map of a subset of cooperative relations among institutions, 2017–2022.

**Figure 4:**
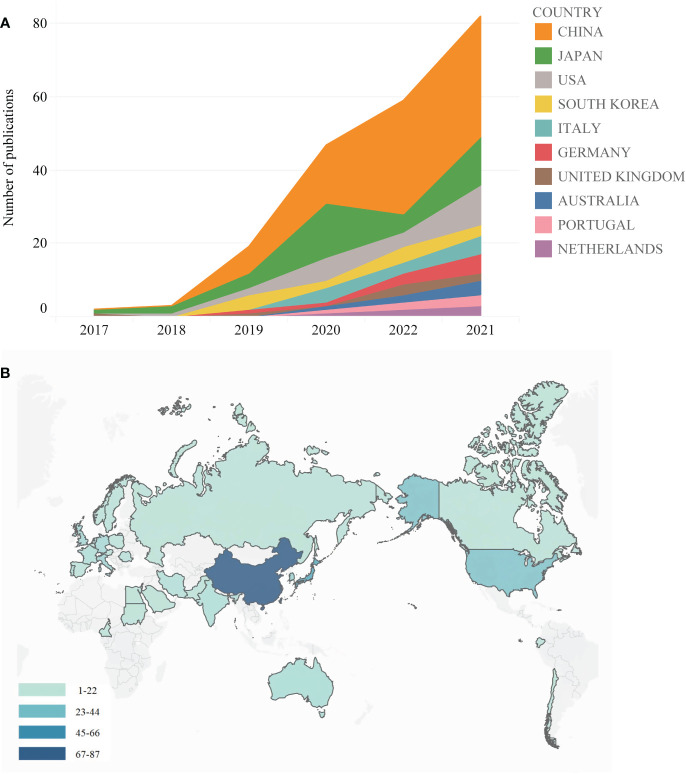
**(A)** The annual number of publications in the top 10 most productive countries from 2017 to 2022. **(B)** A world map displaying the contribution of each country based on publication counts.

A total of 33 countries/regions contributed to the research on the application of AI on GC; **Figure 3A** and **Supplementary Figure S1A** show that there were 34 nodes and 106 connections in the cooperation network between countries/regions, with a density of 0.1889. As shown in **Table 1** and **Figure 4A**, China (87, 47.54%) ranked first in the number of articles published, followed by Japan (n=40, 21.86%), the USA (n=24, 13.11%), South Korea (13, 7.10%), and Italy (n=12, 6.56%). China collaborated with Sudan and Cameroon in 2022 and also with Japan, the USA, Norway, Italy, Germany, and the United Kingdom in 2021. Furthermore, we created a world map that displayed the contribution to this field of each country in **Figure 4B**, using color gradients to show how many publications that these countries have published, and it is clear that most of the articles are published by researchers in East Asia, North America, and other regions.

**Table 1:** The top 10 prolific countries/regions and corresponding institutions.

Ranking	Country	Centrality	Frequency/N (%)	H-index	Average Citation	Institution	Centrality	Frequency/N (%)
1	People’s R China	0.4	87/47.54	16	11.59	Wuhan Univ	0.06	13/7.10
						Sichuan Univ	0.02	8/4.37
						Zhejiang Univ	0	6/3.28
2	Japan	0.09	40/21.86	18.17	14	Univ Tokyo	0.07	11/6.01
						Tada Tomohiro Inst Gastroenterol & Proctol	0.01	11/6.01
						AI Med Serv Inc	0.01	8/4.37
3	USA	0.12	24/13.11	16.3	9	Univ Kansas	0	4/2.19
						Harvard Med Sch	0	2/1.09
						Microsoft Ltd Co	0	2/1.09
4	South Korea	0	13/7.10	13.43	8	Chuncheon Sacred Heart Hosp	0.01	2/1.09
						Gachon Univ	0	2/1.09
						Sungkyunkwan Univ	0	2/1.09
5	Italy	0.18	12/6.56	10.5	8	Nuovo Regina Margherita Hosp	0	2/1.09
						Sapienza Univ Rome	0	2/1.09
						Univ Bologna	0	2/1.09
6	Germany	0.3	10/5.46	8.9	5	Univ Klinikum Augsburg	0	2/1.09
						Alfred Wegener Inst Helmholtz Zentrum Polar & Mee	0	1/0.55
						Berlin Inst Hlth BIH	0	1/0.55
7	England	0.3	7/3.83	38	3	Imperial Coll London	0	1/0.55
						Kings Coll Hosp NHS Fdn Trust	0	1/0.55
8	Australia	0.05	7/3.83	4.86	4	Univ Adelaide	0	3/1.64
						Alfred Hosp	0	1/0.55
						Griffith Univ	0	1/0.55
9	Portugal	0.11	6/3.28	7.83	3	Univ Porto	0	4/2.19
						Portuguese Oncol Inst Porto	0	2/1.09
10	Netherlands	0.07	6/3.28	8.33	3	Eindhoven Univ Technol	0	2/1.09
						Amsterdam UMC Locat Vrije Univ Amsterdam	0	1/0.55
						Amsterdam UMC	0	1/0.55

H-index is frequently used as a quantitative and qualitative indicator of academic output and allows us to have an assessment of the quality and quantity of publications of a nation, a journal, or an institution (27). As shown in **Table 1** and **Figure 4A**, research related to this field in China started later compared with Japan, but the output of articles and the number of achievements in China were much higher than in any other countries, and Chinese centrality was with the highest strength, reaching 0.4. However, the H-index of China (n=16) ranked third, lower than Japan (n=18.17) or the USA (n=16.3), and the average citation of China (n=11.59) was also lower than Japan (14), which indicated that the quality and quantity of output should be enhanced.

Using CiteSpace for organization co-occurrence analysis, we set the node type to Organization, and then the result is shown in **Figure 3B**. We can learn that the cooperation between institutions was roughly divided into four groups. In addition, as presented in **Table 1**, Wuhan University published the largest number of articles among all institutions (13 publications), followed by The University of Tokyo (11 publications) and Tada Tomohiro Inst Gastroenterol and Proctol (11 publications). Furthermore, Wu et al. from Wuhan University, University of Bologna (Italy), University of Oslo (Norway), University of Porto (Portugal), Showa University (Japan), and University of Kansas (USA) conducted a collaborative study to assess the accuracy of endoscopists who use AI validation research framework to identify upper gastrointestinal tumors (UGINs), which demonstrated that the accuracy of endoscopists in identifying UGIN is poor even in frameworks with high prevalence and disease awareness. Future research on AI validation can represent a framework for assessing endoscopist capabilities (28). Then, in a paper published in *Lancet Gastroenterology and Hepatology*, Wu et al. conducted a single-center, tandem, randomized controlled trial to evaluate the effect of AI systems for detecting focal lesions and diagnosing gastric tumors on decreasing the rate of missed gastric tumor diagnoses in clinical practice, and the results showed that the use the AI system during upper gastrointestinal endoscopy significantly reduced the rate of missed diagnosis of gastric tumors. As a result, AI-assisted endoscopy may enhance the detection rate of gastric tumors by endoscopists (29). In a recent study, Wu et al. later proposed a deep-learning-based system, called Endoangel-ME, for diagnosing EGC in magnified image-enhanced endoscopy (M-IEE). Through a multicenter diagnostic study, the results showed that Endoangel-ME could be well applied in clinical settings (30–32). As mentioned above, Japan was the first country to start research in this field, publishing the first research publication in University of Tokyo on AI in GC in 2017. Shichijo Satoki et al. (University of Tokyo) built a convolutional neural network (CNN) and evaluated its capacity to diagnose *Helicobacter pylori* infection, since AI endoscope image in the diagnosis of *H. pylori* gastritis has not been evaluated. Additionally, the findings demonstrated that *H. pylori* gastritis can be diagnosed based on endoscopic image diagnosis to CNN, and this method is time saving and more accurate than the artificial diagnostic endoscopy doctors (33). Background image recognition can be performed by CNN for AI, and deep learning has been significantly improved and increasingly applied to diagnostic imaging in the medical field. Hirasawa Toshiaki et al. (University of Tokyo) thus developed a CNN that can automatically detect GC in endoscopic images, which showed that the constructed CNN system for GC detection can process a large amount of stored endoscopic images in a very short time and has clinically relevant diagnostic capability. Moreover, it may be well adapted to everyday clinical practice to reduce the burden on endoscopists (34). The paper published by Hirasawa Toshiaki et al. has become a highly cited paper with 308 citations. Ryota Niikura, Satoki Shichijo, Toshiaki Hirasawa et al. (University of Tokyo) demonstrated the advantage of AI in the diagnosis of GC by comparing the diagnostic rate of endoscopic GC imaging with that of AI and professional endoscopists, but it did not prove its superiority (35).

#### 3.2.2 Bibliometric analysis of authors and co-authors

The quantity and quality of publications can represent the level of research and contribution of the author in this field. Totally, 201 authors participated in a total of 183 publications. From the perspective of publication count (**Table 2**), Tada (12 publications) had the largest number of articles, followed by Li (10 publications), Yu (10 publications), and Wang (9 publications). Tada from Japan graduated from the University of Tokyo. He is the founder and CEO of AI Medical Service and also serves as the CEO of Tada Tomohiro Inst Gastroenterol and Proctol. Additionally, he had an H-index of 26 and 2,146 citations. We can learn from **Table 1** that these three institutions mentioned above were the top 3 institutions in Japan in terms of the number of articles related on AI applicated in GC. Tada participated in the first research applying AI to GC in 2017 (33) and co-published the highly cited paper (mentioned above in *Section 3.2.1*), with the lead author Hirasawa (34). This highly cited paper has attracted much attention in the field of AI research and has been cited over 337 times up to now. The key authors in a co-citation network of a certain field can be identified by the author co-citation analysis (36). The author with the highest total citations was Hirasawa from the Japan Cancer Research Foundation with 90 total citations, working at the Tomohiro Inst Gastroenterol and Proctol Tada from 2018 to 2021. The major areas of his research were Stomach Cancer, Renal Oncocytoma, Esophageal Cancer, and *Campylobacter* Infection, and his latest findings were mentioned above in *Section 3.2.1* (35). Bray, with the second highest number of co-citations, was also a key author in this field, and he was branch head cancer surveillance in the International Agency for Research on Cancer with specialist in descriptive epidemiology of cancer, time trends and predictions, and cancer registries.

**Table 2:** The top 10 most productive authors and top 10 co-cited authors.

Ranking	Author	Count	Centrality	H-index	Country	Co-cited author	Count	Centrality	H-index	Country
1	Tada T	12	0.01	26	Japan	Hirasawa T	90	0.03	26	Japan
2	Li Y	10	0.05	25	People’s R China	Bray F	61	0	108	France
3	Yu H	10	0.01	33	People’s R China	Zhu Y	59	0.03	12	People’s R China
4	Wang Y	9	0.02	14	People’s R China	Wu LL	56	0.04	15	People’s R China
5	Wu L	8	0.01	15	People’s R China	Horiuchi Y	46	0.03	17	Japan
6	He X	8	0.01	11	People’s R China	Li L	43	0.01	15	People’s R China
7	Zhang Y	7	0.06	12	People’s R China	Mori Y	42	0.01	29	Norway
8	Liu J	7	0.1	16	People’s R China	Shichijo S	39	0.11	21	Japan
9	Fujisaki J	6	0.01	28	Japan	Horie Y	39	0.04	17	Japan
10	Kim J	6	0.01	18	South Korea	Kanesaka T	39	0.07	/	/

**Figure 5:**
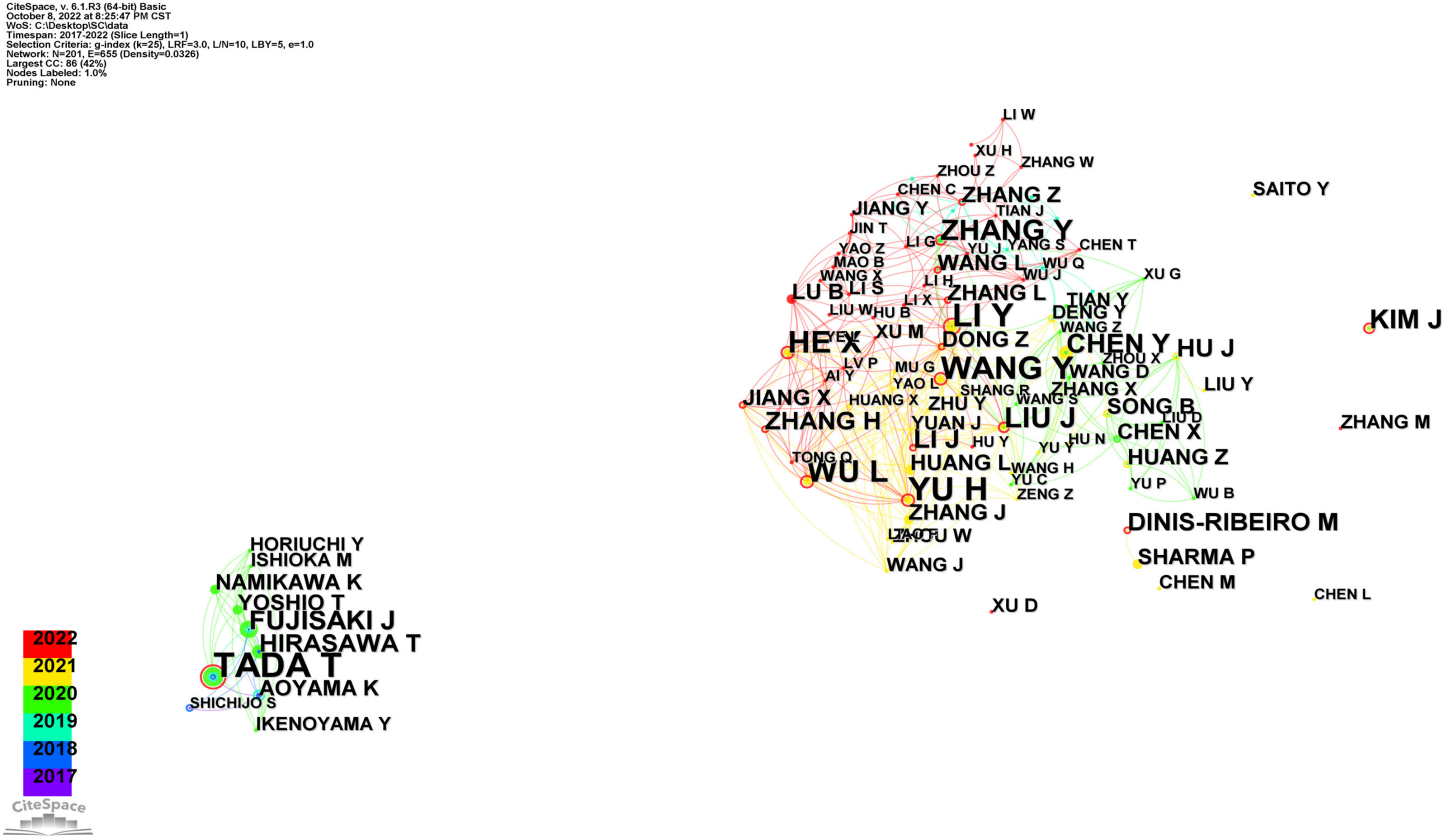
The network of authors, 2017–2022.

Researchers that focus on research have abundant and unique professional knowledge, and cooperation between them can enhance communication and productivity of a certain research subject. In addition, researchers can learn existing partnerships and develop potential cooperative subjects by analyzing the co-authorship of authors. Therefore, we used CiteSpace to make a network diagram of author cooperation, from which we can observe that author cooperation was roughly divided into two groups. The smaller group included Japanese authors and was mainly led by Tada and Hirasawa, while the larger group included Chinese authors led by Li, Yu, Wang et al. (**Figure 5** **)**.

#### 3.2.3 Bibliometric analysis of the higher-impact journals

In this study, all these publications related to AI applicated in GC were distributed in 94 academic journals and co-cited journals. **Table 3** summarizes the basic information on the top 20 journals and co-cited journals. The impact factor (IF) is frequently used as an indicator of the significance of a journal to its field of a 2-year moving average citation of a journal, which was first introduced by Eugene Garfield, the founder of the Institute for Scientific Information (37). As shown in **Table 3**, we can learn that 45% of journals and 55% of co-cited journals belong to Q1. The journal *Gastrointestinal Endoscopy* (21 publications; IF 2022, 9.189; Q1) with the second highest number of citations (115 citations, 11.5%) had the highest number of outputs on AI applicated in GC, followed by *World Journal of Gastroenterology* (13 publications, 7.1%; IF 2022, 5.715; Q2) and *Digestive Endoscopy* (12 publications, 6.6%; IF 2022, 5.779; Q2). In addition, the journal *Gastric Cancer* (133 citations; IF 2022, 8.171; Q1) had the highest citations among them, followed by *Gastrointestinal Endoscopy* (115 citations; IF 2022, 9.189; Q1), *Gut* (109 citations; IF 2022,27.827; Q1), and *Endoscopy* (106 citations; IF 2022, 9.508; Q1). The frequency of being co-cited, which reflects whether a journal has a huge impact on a particular research field, determines the influence of journals. Therefore, the main research direction of these journals was *Gastrointestinal Endoscopy*, *Gastric Cancer*, and *Endoscopy*. Furthermore, it is worth mentioning that *Gastrointestinal Endoscopy* ranked first in journal and second in co-cited journal, which indicated that it had an absolute influence on AI applicated in GC.

**Table 3:** The top 20 journals and co-cited journals.

Ranking	Journal	Output	IF	JCR	Co-cited journal	Citation	IF	JCR
1	Gastrointestinal Endoscopy	21	9.189	Q1	Gastric Cancer	133	8.171	Q1
2	World Journal of Gastroenterology	13	5.715	Q2	Gastrointestinal Endoscopy	115	9.189	Q1
3	Digestive Endoscopy	12	5.779	Q2	Gut	109	27.827	Q1
4	Diagnostics	9	4.129	Q2	Endoscopy	106	9.508	Q1
5	Frontiers in Oncology	8	6.122	Q2	Gastroenterology	97	29.175	Q1
6	Endoscopy	7	9.508	Q1	Digestive Endoscopy	91	5.779	Q2
7	Cancers	5	6.886	Q1	CA—A Cancer Journal for Clinicians	90	334.259	Q1
8	Frontiers in Medicine	5	5.493	Q2	World Journal of Gastroenterology	89	5.715	Q2
9	Gastric Cancer	5	8.171	Q1	Scientific Reports	82	5.516	Q2
10	BMC Gastroenterology	4	3.23	Q4	Journal of Gastroenterology and Hepatology	65	4.319	Q2
11	Computational and Mathematical Methods in Medicine	4	2.887	Q2	Endoscopy International Open	65	0.77	Q2
12	Journal of Gastroenterology and Hepatology	4	4.319	Q2	eBioMedicine	65	10.481	Q1
13	Journal of Medical Internet Research	4	7.69	Q1	Nature	59	63.58	Q1
14	Surgical Endoscopy and Other Interventional Techniques	4	3.796	Q2	Lancet Oncology	59	49.204	Q1
15	Best Practice Research Clinical Gastroenterology	3	4.875	Q4	PLOS One	58	4.069	Q2
16	eBioMedicine	3	10.481	Q1	Digestive Diseases and Sciences	57	3.522	Q3
17	Journal of Clinical Oncology	2	38.801	Q1	Surgical Endoscopy and Other Interventional Techniques	54	3.796	Q2
18	Annals of Translational Medicine	2	4.263	Q3	JAMA—Journal of the American Medical Association	53	101.13	Q1
19	Archives of Computational Methods in Engineering	2	8.967	Q1	New England Journal of Medicine	49	125.115	Q1
20	Chinese Medical Journal	2	3.81	Q1	Journal of Clinical Medicine	45	5.098	Q2

#### 3.2.4 Analysis of co-cited reference

It is an efficient method to assess the progress and trace the developmental frontiers of any research field using reference co-citation analysis (38). The top 10 co-cited references related to our research are presented in **Table 4**. The paper titled “Application of AI using a convolutional neural network for detecting gastric cancer in endoscopic images” published by Hirasawa in *Gastric Cancer* in 2018 ranked first with a total number of citations of 89. The second is “Global cancer statistics 2018: GLOBCAN estimates of incidence and mortality worldwide for 36 satellites in 185 countries” published by Bray in *CA—Cancer J Clin* (60 citations), followed by “Application of convolutional neural network in the diagnosis of the invasion depth of gastric cancer based on conventional endoscopy” (57 citations). Four of the top 5 papers were highly cited, which showed that there was a certain relationship between citation times and co-citation times.

**Table 4:** The top 10 co-cited reference.

Ranking	Title	Journal	First Author	Publication year	Total citations
1	Application of AI using a convolutional neural network for detecting gastric cancer in endoscopic images	*GASTRIC CANCER*	Hirasawa T	2018	89
2	Global cancer statistics 2018: GLOBCAN estimates of incidence and mortality worldwide for 36 cancers in 185 countries	*CA-CANCER J CLIN*	Bray F	2018	60
3	Application of convolutional neural network in the diagnosis of the invasion depth of gastric cancer based on conventional endoscopy	*GASTROINTEST ENDOSC*	Zhu Y	2019	57
4	Convolutional neural network for the diagnosis of early gastric cancer based on magnifying narrow band imaging	*GASTRIC CANCER*	Li L	2020	43
5	A deep neural network improves endoscopic detection of early gastric cancer without blind spots	*ENDOSCOPY*	Wu LL	2019	41
6	The diagnostic outcomes of esophageal cancer by AI using convolutional neural networks	*GASTROINTEST ENDOSC*	Horie Y	2019	39
7	Computer-aided diagnosis for identifying and delineating early gastric cancers in magnifying narrow-band imaging	*GASTROINTEST ENDOSC*	Kanesaka T	2018	38
8	Convolutional Neural Network for Differentiating Gastric Cancer from Gastritis Using Magnified Endoscopy with Narrow Band Imaging	*DIGEST DIS SCI*	Horiuchi Y	2020	35
9	Real-time AI for detection of upper gastrointestinal cancer by endoscopy: a multicentre, case-control, diagnostic study	*LANCET ONCOL*	Luo HY	2019	34
10	Application of Convolutional Neural Networks in the Diagnosis of Helicobacter pylori Infection Based on Endoscopic Images	*EBIOMEDICINE*	Shichijo S	2017	32

### 3.3 Research topic and hotspots analysis—analysis of keywords and keywords with citation burst

**Figure 6:**
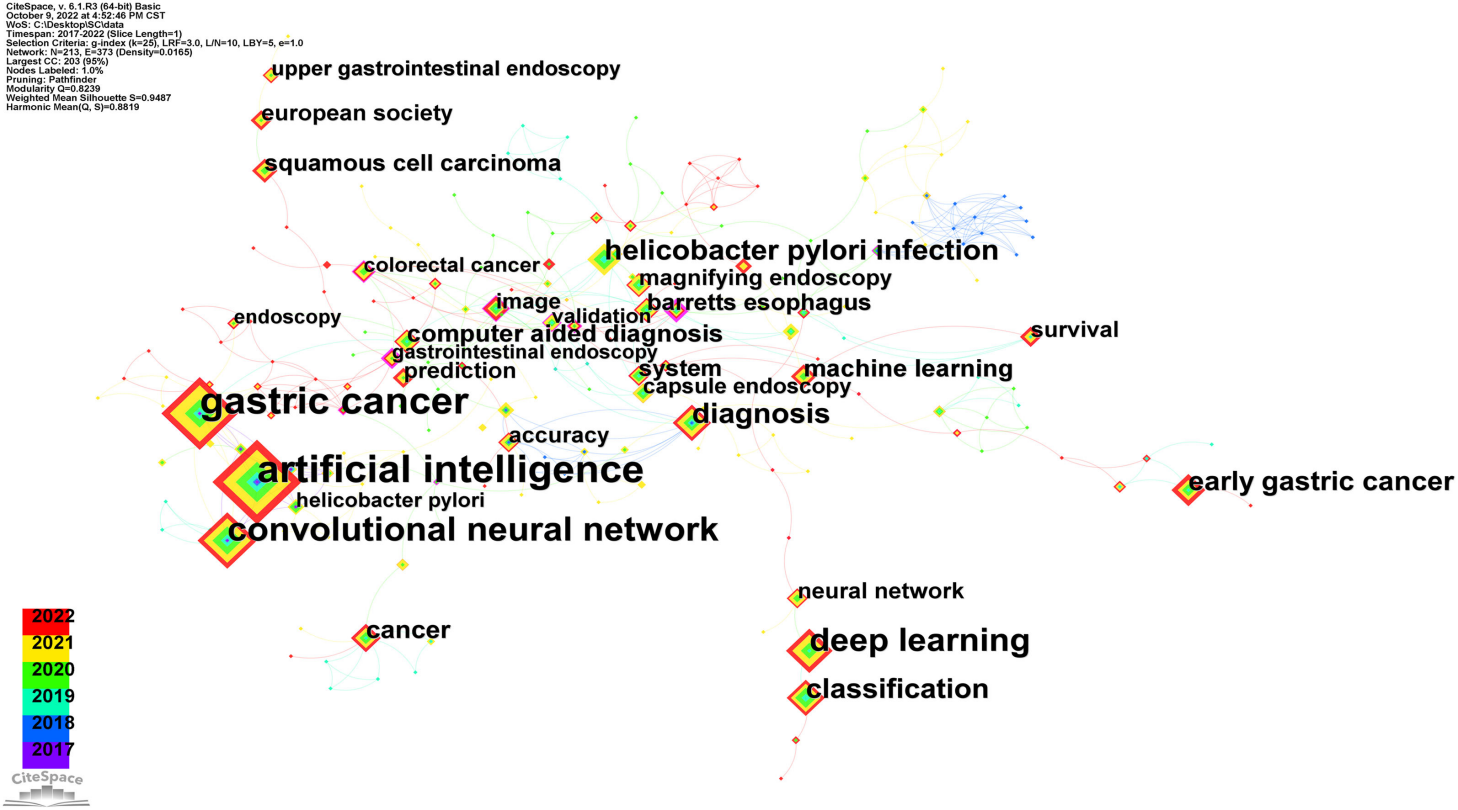
The keyword visualization map.

**Figure 7:**
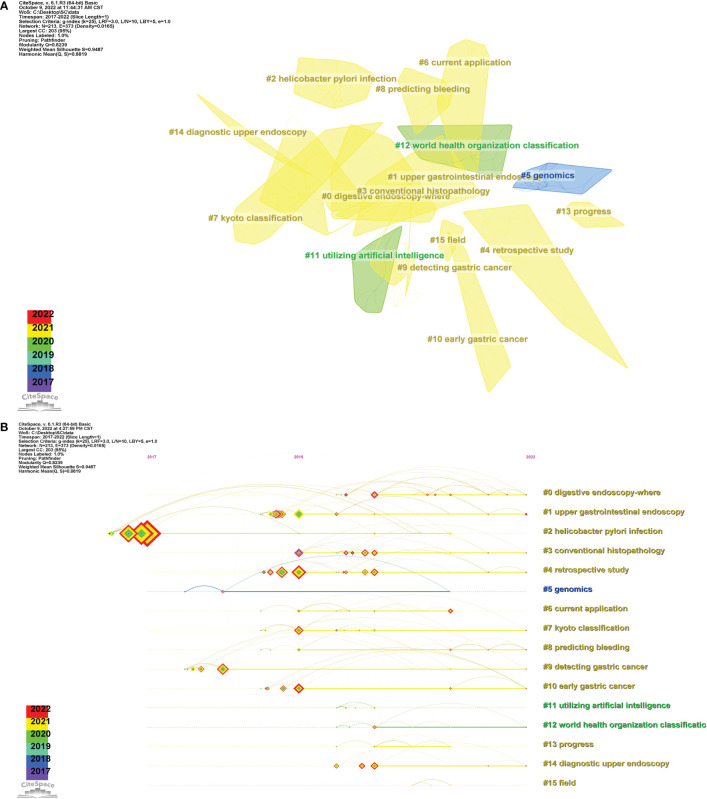
**(A)** The cluster view map of keyword. **(B)** The cluster timeline view map of keywords analysis.

Keywords are the crystallization of the text content of an article, which is with a high generalization and reflection ability in a research field and can directly point to the center of the text. Therefore, keywords with high frequency are often used to present the hot issues in a research field and reflect the research hotspots in a certain period of time from a macroperspective. In order to describe the research status and hotspots of AI in the field of GC, the knowledge map of keyword co-occurrence was obtained using CiteSpace (**Figure 6** **)**. The knowledge map of keyword co-occurrence was obtained through CiteSpace analysis and presented in two forms: Cluster View (**Figure 7A**) and Timeline View (**Figure 7B**).

Centrality represents the degree of importance of the node in the network co-occurrence, so keywords with high centrality may be regarded as the research hotspots in a certain field over a period of time (24). As mentioned above, the larger the node is in the keyword visualization map, the more frequent the keyword co-occurrence is. Additionally, the thickness of the lines represents the co-occurrence intensity between nodes, and the thicker the line is, the greater the co-occurrence intensity is. Therefore, the higher the co-occurrence frequency and centrality of node are, the more important the keyword in the research field is. The node of “AI” is the largest, followed by “gastric cancer,” “convolutional neural network,” and “deep learning” through CiteSpace and VOSviewer (**Figure 6**; **Supplementary Figure S1B**). According to the data in **Table 5**, the number of their appearances is 118, 101, 61, 48, 29, and 28, respectively.

**Table 5:** Top 20 keywords.

Ranking	Keyword	Count	Centrality	Year
1	AI	118	0	2017
2	gastric cancer	101	0.14	2017
3	convolutional neural network	61	0.05	2017
4	deep learning	48	0.05	2019
5	Diagnosis	29	0.11	2018
6	*Helicobacter pylori* infection	28	0.06	2019
7	classification	27	0.04	2019
8	early gastric cancer	23	0.02	2019
9	cancer	18	0.12	2019
10	machine learning	17	0	2020
11	computer aided diagnosis	16	0.14	2019
12	Barrett’s esophagus	14	0.14	2019
13	system	14	0.19	2019
14	squamous cell carcinoma	14	0.05	2020
15	magnifying endoscopy	13	0	2019
16	European Society	13	0.04	2020
17	neural network	13	0.09	2020
18	prediction	13	0	2020
19	accuracy	13	0.1	2018
20	survival	13	0.12	2019

Finally, we gained the clustering function in **Figure 7A**. It is worth mentioning that the modularity value (Q-value) and mean silhouette value (S-value) are two important indicators to evaluate the significance of community structure, and a clustering with a Q > 0.3 and S > 0.7 is significant. There were 16 different clusters in the network map, and the Q-value (0.8239) and weighted mean silhouette (0.9487) demonstrated the reasonableness of this network (39). From **Figure 7A** and **Table 6**, it can be observed that “upper gastrointestinal endoscopy” #0 and “*Helicobacter pylori* infection” #1 were the largest cluster, followed by “conventional histopathology” #2, “retrospective study genomics” #3, and “current application” #4.

**Table 6:** The clusters information of keywords.

ClusterID	Label	Size	Silhouette	Mean(year)
#0	upper gastrointestinal endoscopy	22	0.948	2021
#1	helicobacter pylori infection	22	0.925	2020
#2	conventional histopathology	18	0.958	2019
#3	retrospective study	16	0.891	2020
#4	Genomics	16	0.924	2020
#5	current application	16	0.93	2018
#6	Kyoto classification	13	0.936	2020
#7	predicting bleeding	12	0.981	2019
#8	detecting gastric cancer	12	0.954	2020
#9	early gastric cancer	11	0.994	2019
#10	utilizing AI	10	1	2020
#11	World Health Organization classification	9	0.958	2020
#12	progress	8	0.977	2020
#13	diagnostic upper endoscopy	7	0.972	2020
#14	field	6	0.955	2020
#15	upper gastrointestinal endoscopy	5	0.985	2021

In order to further analyze the keywords of AI applicate in GC, a Timeline View analysis was conducted. For time clustering, click “Find Clusters,” then “LLR,” and select “Timeline View” in Layout last, and the result is finally shown in **Figure 7B**. The evolution pace of each cluster by time could be observed to further explore the key research contents in this field from a microperspective. In **Figure 7B**, there remained a total of 16 clustering, numbered from 0 to 15. Moreover, the distance from left to right or from top to bottom of every clustering and the size of the color line load point represent the appearing time and end time of each clustering, and color curve represents cluster label word co-occurrence relation between the different colors. We can see that the research focus has shifted from “genomics” (5), “utilizing AI” (11), and “world health organization classification” (12) to “upper gastrointestinal endoscopy” (1), “*Helicobacter pylori* infection” (2), “conventional histopathology” (3), “retrospective study” (4), “current application” (5), “Kyoto classification” (6), and “predicting bleeding” (7).

**Figure 8:**
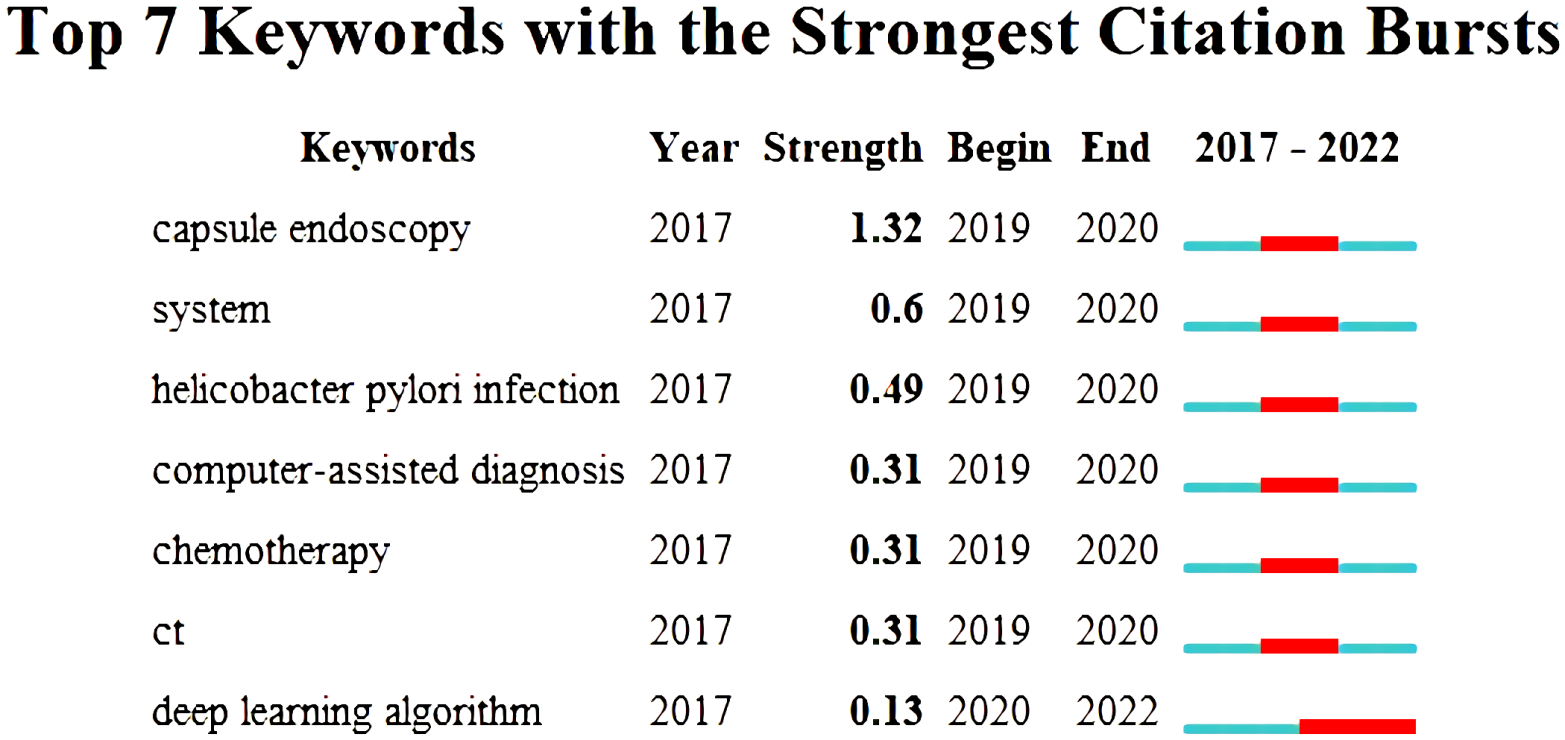
The top 7 keywords with the strongest citation burst.

Additionally, burst detection, an algorithm developed by Kleinberg (40), is an effective analytical tool for capturing the turning point in keywords or references popularity over a specified period. **Figure 8** shows the top 7 keywords with the strongest keyword outbreak. The blue line represents the time interval, and the red line represents the duration of the outbreak. The first six keywords started to break out in 2019, and the end time was 2020, while the “deep-learning algorithm” started to break out in 2020 and continues till now, which indicated that this research topic has attracted continuous attention in recent years and might be the trend of research on AI application in GC.

## 4 Discussion

### 4.1 General information

In the present study, we performed a systematic literature search of the WoS databases for articles published from 2017 to 2022 about AI applicated in GC, a total of 183 publications in this research field was retrieved and plotted. As shown in **Figure 2B**, due to the accelerated development and research of machine learning and artificial neural networks, great breakthroughs have taken place in AI in the 1990s. Meanwhile, the number of publications on AI has been gradually increasing, and the application of AI in medicine has also increased, but it is still relatively small. The publication of the relevant papers was an important inflection point in 2016, the annual growth rate from 2016 to 2017 was the highest, and the first application of AI in the field of GC in the database was published in 2017. Because an important event occurred in this year, Alpha Go defeated the world Go master Lee Sedol, pushing the high tide of AI development to a new level (41). Meanwhile, the world’s major economic powers have accelerated the deployment of AI, which laid a firm foundation for new breakthroughs in the field of AI on a global scale causing the increase in investment in the AI industry and various policies to encourage the development of AI. The application of AI in the field of medicine has also developed accordingly, and the number of publications has increased annually, indicating that this topic has received significant attention. Additionally, 2017 was the year when AI was first universally recognized in the world. In this year, Alpha Go Zero achieved self-renewal and upgrading through deep learning, constantly surpassing itself and beating Alpha Go (42). Moreover, Watson, an AI developed by IBM, analyzes and interprets massive medical data and literature through machine learning and then proposes treatment plans, which highly coincide with doctors’ treatment recommendations (retrieved 14 October 2022, https://en.wikipedia.org/wiki/IBM_Watson). Xiaoice is an AI system developed by Microsoft, and through self-study on modern poetry, the first AI-authored collection of poems—The Sunshine Lost Windows—was published by Xiaoice in May 2017 (retrieved 14 October 2022, https://en.wikipedia.org/wiki/Xiaoice). Research on AI in the field of GC has also received corresponding attention; the annual average growth rate of relevant articles reached 264.11% particularly in 2021(**Figure 2A**). Although the overall research base of AI in the field of GC is relatively small, according to the current trend, it indicates that it will be a research hotspot in the future and will continue to receive attention.

Using visual analysis of the distribution of countries and institutions enables us to better understand which countries and institutions are the main contributors to this field. As shown in **Table 1** and **Figure 4A**, we could see that China ranked first in the number of articles published. Moreover, institutional distribution was generally consistent with the country distribution, and the institution with the largest output of publications was Wuhan University, while University of Tokyo and Tada Tomohiro Inst Gastroenterol and Proctol followed. The explanation for this could be due to the fact that East Asia is a region with highest incidence of GC (3). Although China has made a late start in this field compared with Japan, but its pace of development is staggering. In addition, Chinese centrality was with the highest strength, reaching 0.4. The above results were attributed to the Chinese government’s significant support for AI applications. Unfortunately, when the theme distribution of the H-index and the average citation were examined, China ranked third and second, respectively, indicating that the impact of output should be improved. However, with the rapid development, China will potentially take the leading and dominant position in this field. As shown in **Figures 3A, B**, although countries possess their cooperation networks, the connection between countries is sparse. From the perspective of research institutions, we could learn that the cooperation between institutions was roughly divided into four groups; there is substantially less collaboration and exchange of achievements across groups. Nevertheless, the objective situation is that relatively few countries are currently involved in research related to the application of AI in GC worldwide. These situations hinder the development of this research area. Meanwhile, this also means that there is a large research gap that could be further explored and paid more attention in this field. Therefore, it is strongly suggested to strengthen the international exchanges and collaborations between countries and institutions to promote the research and development of AI in GC.

The emergence of academic journals always plays a significant role not only in the presentation of research results and science communication of scientists but also in our demand for academic knowledge. Publications serve as carriers for achievements in scientific research, and effective scientific communication requires the research results in an international peer-reviewed journal. Therefore, through the analysis of the distribution of journal sources, researchers can rapidly locate the most appropriate journals for their papers (43). Journals and Co-cited Journals Analysis (**Table 3**) showed that 45% of journals and 55% of co-cited journals were located in the Q1 JCR region. The journal *Gastrointestinal Endoscopy* had the highest number of outputs and the second highest number of citations for AI applied to GC, indicating that the journal has a significant influence in the field of AI in GC. In addition, the top journal in the field of gastric cancer, *Gastric Cancer*, had the highest citations among them. The IF of these journals could reflect the importance and priority of AI to some extent. These high-quality journals have a significant influence on their respective academic fields, which further indicate AI applicated in GC as an important research direction for GC. In terms of the category of subject distribution, besides medical-related journals, it also includes one journal of engineering and technical, *Archives of Computational Methods In Engineering*. This indicates that the application of AI in GC is an interdisciplinary field, and its development requires multidisciplinary cooperation.

Generally, the authors who are frequently cited are regarded to have more impact than those who are less cited, and authors who are jointly cited probably focus on the relevant research field. From the perspectives of author contributions and co-cited authors (**Table 2**), the author with the most published articles in this field was Tada with 12 published articles, whose research focused on AI Medical Service. Moreover, Tada participated in the first research applying AI to GC in 2017 [26]. When co-cited authors were taken into account, Hirasawa was the most frequently co-cited author at 90 times, while Shichijo had the highest centrality. It should be noted that almost all of the top 10 active and co-cited authors were from Asia, implying that Asian researchers have played an important role and made significant contributions to AI in GC research. Furthermore, after comprehensive analysis of the data of the network diagram of author cooperation, we found that the author cooperation can be roughly divided into two groups, represented by Tada, Hirasawa, and Li, Yu, and Wang, respectively (**Figure 5**). The results indicate that Tada, Hirasawa, and Li are accomplished authors in this field and have a significant influence on other authors, and their teams would be excellent potential collaborators for researchers.

### 4.2 Knowledge base

Reference co-citation analysis is a valuable technique to assess the evolution and trace the developmental frontiers of a research field (38). The knowledge base is a collection of co-cited references (21). The top 10 co-cited references related to our research are presented in **Table 4**, which includes nine clinical trials and one epidemiological study. Four of the top 5 papers were highly cited papers, which showed that there was a certain relationship between citation times and co-citation times.

Almost all clinical trials were focused on the diagnosis and differential diagnosis of AI in GC, with the exception of one study of CNNs in the endoscopic imaging diagnosis of *H. pylori* infection. Shichijo (33) first constructed a CNN in 2017 to evaluate its ability to diagnose *H. pylori* infection. Compared with the artificial diagnostic endoscopists, the accuracy is higher, and it takes less time. Among the most frequently co-cited references related to application of AI in GC was a paper published by Hirasawa in *Gastric Cancer* in 2018, which was the most cited paper (89 citations). The authors constructed a CNN-based diagnostic system and used it for an independent test set of 2,296 gastric images collected from 69 consecutive patients with 77 GC lesions. The results showed that the CNN system could be used to detect GC with a clinically relevant diagnostic ability (34). Simultaneously, another clinical trial conducted by Kanesaka et al. developed a computer-aided diagnosis (CAD) system to assist endoscopists in identifying and diagnosing EGCs (44). The diagnostic performance revealed high sensitivity and accuracy, illustrating the great potential in real-time diagnosis and delineation of EGCs in M-NBI images. One year later, Zhu et al. published the results of a clinical trial using a CNN-CAD system to determine the invasion depth of GC with high accuracy and specificity and thereby could reduce unnecessary gastrectomy (45). Similarly, Wu et al. presented the results of a novel deep convolution neural network (DCNN) to detect EGCs. Compared to endoscopists, the system could detect and localize EGC more accurately. Moreover, it could significantly actively track suspected cancerous lesions and monitor blind spots during esophagogastroduodenoscopy (46). Another clinical trial published by Horie et al. evaluated the sensitivity and accuracy of the CNN system in detecting esophageal cancer. The results showed that 1,118 test images were analyzed in 27 s, with a sensitivity of 98% in detecting esophageal cancer cases and an accuracy of 98% in identifying superficial esophageal cancer and advanced esophageal cancer (47). The only one multicenter case–control diagnostic study was published in *Lancet Oncol* by Luo’s group (48). The authors successfully constructed the Gastrointestinal Artificial Intelligence Diagnostic System (GRAIDS), and the result proved that GRAIDS achieved high diagnostic accuracy and sensitivity in upper gastrointestinal cancers. The two clinical studies published in 2020 focused on CNN system in the diagnosis of EGC and in the differential diagnosis of gastritis and GC observed by magnifying endoscopy with narrow band imaging(M-NBI) (49, 50). Bray et al. reported on cancer incidence and mortality of 36 cancers in 185 countries/regions worldwide in *CA—A Cancer Journal for Clinicians*, which contributed notable epidemiological data on GC (3).

In summary, there is no doubt that the top 10 most co-cited references provide an important foundation for the development of this field. The emphasis of these studies mainly includes the application of AI in the early diagnosis and differential diagnosis of GC to provide accurate guidance and support for clinical therapy. It is gratifying to see that some breakthroughs have been achieved in the research of the application of AI in GC. The analysis of co-cited reference allows us to better understand the evolution of the application of AI in GC.

### 4.3 The analysis of research hotspots

Keywords with high frequency are often used to present the hot issues in a research field and reflect the research hotspots in a certain period of time from a macroperspective. To understand the research hotspots and frontiers of AI in GC, we visualized the keywords in **Figure 6**. The more representative keywords include AI, gastric cancer, convolutional neural network, deep learning, diagnosis, *Helicobacter pylori* infection, which indicate that these topics are research hotspots in this field. Currently, the application of AI in GC is mainly for diagnostic and differential diagnosis, and convolutional neural networks and deep learning are the most commonly used methods.

Furthermore, the knowledge map of keyword co-occurrence was obtained through CiteSpace analysis. With the help of the clustering function, the whole network map could be sorted into some different clusters, and studies in the same cluster might have similar research themes. The clusters were named by extracting nominal terms as labels from the titles of the cited articles, and the log-likelihood ratio (LLR) algorithm is applied as the extraction method. As shown in **Figure 7A** and **Table 6**, the cluster view of the keywords revealed that “upper gastrointestinal endoscopy” #0 and “helicobacter pylori infection” #1 were the largest cluster, indicating that the application of AI in endoscopy may be a mature and important theme in the AI research field. Simultaneously, a Timeline View analysis was conducted to observe the evolution pace of each cluster by time and to further explore the key research contents in this field from a microperspective. We can clearly observe in **Figure 7B** that the research focus has shifted; after a period of technical reserve based on theoretical research and basic applications, AI-related research in GC has gradually shifted toward clinical applications. AI has been widely applied in the field of endoscopy, pathology, and radiomics, achieving inspiring results and providing accurate guidance and support for clinical therapy decisions.

Additionally, since burst detection was an effective analytic tool to capture the dramatic increases in references or keywords popularity within a specified period, it therefore served as an important indicator of research hotspots or research frontiers over time. The evolution of the burst keywords over the last 5 years is shown in **Figure 8**. According to the ranking by burstiness strength, we observed that the top 6 keywords started to burst in 2019, and the end time was 2020. More importantly, the “deep-learning algorithm” started to burst in 2020 and continues till now, which indicated that this research topic has attracted continuous attention in recent years and might be the trend of research on AI application in GC. More importantly, the “deep-learning algorithm” started to burst in 2020 and continues till now. Deep learning was widely applied in the clinical diagnosis, therapy, and prognosis prediction of GC (9, 51–54). In a retrospective multi-institutional study enrolling 2,320 patients, Jiang et al. developed a multitask deep-learning model that could accurately predict peritoneal recurrence and survival in GC patients by using preoperative CT images (51). Another retrospective multicenter cohort study suggested that deep-learning-based classifiers for microsatellite instability and EBV-positive detection could be used as convenient and inexpensive predictive biomarkers for immunotherapy in GC, which has significant implications for the comprehensive therapy of GC (52). Moreover, a deep learning radiomic nomogram (DLRN) was built to determine the number of lymph node metastasis (LNM) in the locally advanced gastric cancer (LAGC). The results showed its excellent predictive value and provide baseline information for individualized treatment of LAGC (9). These studies illustrated that this research topic has attracted continuous attention in recent years and might be the trend of research on AI application in GC.

AI approaches such as machine learning and deep learning allows extracting quantitative features non-invasively from digital medical image for histological classification, tumor staging, therapy response, and prognosis. With the digital innovations including tele-health and 5G, AI application will become an integral part of GC research. However, the development of AI in GC is limited by issues such as the generality of models built by AI technology and the security of AI system data, which is the future direction of AI in GC.

According to the above analysis, our bibliometric study conducted a systematic analysis of the basic situation, research hotspots, and trends in AI in the field of GC from a visualization perspective. Therefore, the results of the bibliometric study were objective and accurate, which could provide a comprehensive guide for clinicians and academics working in this field. Given the important role of AI technology in GC and its significant advantages in the clinical diagnosis, therapy, and prognosis prediction of GC, without a doubt, the application of AI in the field of GC is a current research hotspot and will be a major research direction for scholars in the following years.

## 4 Limitation

The bibliometric analysis yielded a great deal of valuable information but inevitably had some limitations. Although the WoSCC database is one of the most comprehensive, systematic, and authoritative databases widely used for bibliometric analysis and visualization of scientific literature, we only used data from the WoSCC database and excluded data from other databases, which may have affected the results of the study. Additionally, the rigorous retrieval strategy and the restriction to articles published in English can also result in the loss of some data. Nevertheless, most of the publications on AI applied to GC are included in this study and can represent the actual research hotspots, evolutionary processes, and trends in this field.

## 5 Conclusion

AI possesses essential research value and application prospects in GC, especially showing great advantages in the clinical diagnosis, therapy, and prognosis prediction of GC. Research on AI in GC is increasing exponentially, highlighted by the outstanding contributions of China, Japan, and the USA to the development of this field. Current research hotspots focus on the application of AI in the diagnosis and differential diagnosis and staging to provide accurate guidance and support for clinical treatment, and convolutional neural networks and deep learning are the most commonly used methods. Meanwhile, deep-learning algorithm has attracted continuous attention in recent years and will serve as the focus of future research. Therefore, it is urgent to strengthen the cooperation and communication between countries and institutions to promote development in this field and benefit more patients with GC.

## Data availability statement

The original contributions presented in the study are included in the article. Further inquiries can be directed to the corresponding authors.

## Author contributions

ZJ and ZL designed this study. GZ, JS and ZF performed the search and prepared the paper. WZ, PH, LL, YZ, XS and YW re-checked data. YC, ZJ and ZL revised and reviewed the manuscript analysis. All authors contributed to the article and approved the submitted version.

## Supplementary material

Supplementary Figure 1
**(A)** Pictorial representation of countrywide collaboration using the country collaboration map.(**B)** The keyword visualization map by VOSviewer.

## References

[1] Van Cutsem E Sagaert X Topal B Haustermans K Prenen H. Gastric cancer. Lancet (London England) (2016) 388(10060):2654–64. doi: 10.1016/s0140-6736(16)30354-3 27156933

[2] GBD 2017 Stomach Cancer Collaborators. The global, regional, and national burden of stomach cancer in 195 countries, 1990-2017: a systematic analysis for the global burden of disease study 2017. Lancet Gastroenterol Hepatol (2020) 5(1):42–54. doi: 10.1016/s2468-1253(19)30328-0 31648970 PMC7033564

[3] Bray F Ferlay J Soerjomataram I Siegel RL Torre LA Jemal A. Global cancer statistics 2018: GLOBOCAN estimates of incidence and mortality worldwide for 36 cancers in 185 countries. CA: Cancer J Clin (2018) 68(6):394–424. doi: 10.3322/caac.21492 30207593

[4] Sung H Ferlay J Siegel RL Laversanne M Soerjomataram I Jemal A. Global cancer statistics 2020: GLOBOCAN estimates of incidence and mortality worldwide for 36 cancers in 185 countries. CA: Cancer J Clin (2021) 71(3):209–49. doi: 10.3322/caac.21660 33538338

[5] Wang FH Zhang XT Li YF Tang L Qu XJ Ying JE. The Chinese society of clinical oncology (CSCO): Clinical guidelines for the diagnosis and treatment of gastric cancer, 2021. Cancer Commun (London England) (2021) 41(8):747–95. doi: 10.1002/cac2.12193 PMC8360643 34197702

[6] Oda I Oyama T Abe S Ohnita K Kosaka T Hirasawa K. Preliminary results of multicenter questionnaire study on long-term outcomes of curative endoscopic submucosal dissection for early gastric cancer. Digestive Endoscopy Off J Japan Gastroenterological Endoscopy Soc (2014) 26(2):214–9. doi: 10.1111/den.12141 23826719

[7] Katai H Ishikawa T Akazawa K Isobe Y Miyashiro I Oda I. Five-year survival analysis of surgically resected gastric cancer cases in Japan: a retrospective analysis of more than 100,000 patients from the nationwide registry of the Japanese gastric cancer association (2001-2007). Gastric Cancer Off J Int Gastric Cancer Assoc Japanese Gastric Cancer Assoc (2018) 21(1):144–54. doi: 10.1007/s10120-017-0716-7 28417260

[8] Ajani JA D'Amico TA Bentrem DJ Chao J Cooke D Corvera C. Gastric cancer, version 2.2022, NCCN clinical practice guidelines in oncology. J Natl Compr Cancer Network JNCCN (2022) 20(2):167–92. doi: 10.6004/jnccn.2022.0008 35130500

[9] Dong D Fang MJ Tang L Shan XH Gao JB Giganti F. Deep learning radiomic nomogram can predict the number of lymph node metastasis in locally advanced gastric cancer: an international multicenter study. Ann Oncol Off J Eur Soc Med Oncol (2020) 31(7):912–20. doi: 10.1016/j.annonc.2020.04.003 32304748

[10] Seevaratnam R Cardoso R McGregor C Lourenco L Mahar A Sutradhar R. How useful is preoperative imaging for tumor, node, metastasis (TNM) staging of gastric cancer? a meta-analysis. Gastric Cancer Off J Int Gastric Cancer Assoc Japanese Gastric Cancer Assoc (2012) 15 Suppl 1:S3–18. doi: 10.1007/s10120-011-0069-6 21837458

[11] Lee DH Kim SH Joo I Han JK. CT perfusion evaluation of gastric cancer: correlation with histologic type. Eur Radiol (2018) 28(2):487–95. doi: 10.1007/s00330-017-4979-5 28779403

[12] Ting DSW Carin L Dzau V Wong TY. Digital technology and COVID-19. Nat Med (2020) 26(4):459–61. doi: 10.1038/s41591-020-0824-5 PMC7100489 32284618

[13] Bi WL Hosny A Schabath MB Giger ML Birkbak NJ Mehrtash A. Artificial intelligence in cancer imaging: Clinical challenges and applications. CA: Cancer J Clin (2019) 69(2):127–57. doi: 10.3322/caac.21552 PMC6403009 30720861

[14] Liu DY Gan T Rao NN Xing YW Zheng J Li S. Identification of lesion images from gastrointestinal endoscope based on feature extraction of combinational methods with and without learning process. Med image Anal (2016) 32:281–94. doi: 10.1016/j.media.2016.04.007 27236223

[15] Dracos A Cognetti G. [Scientific literature: bibliometric and bibliographic indicators as integrative criteria for an objective evaluation of research activity]. Annali dell'Istituto superiore di Sanita (1995) 31(3):381–90. 8712583

[16] Chen C. Searching for intellectual turning points: progressive knowledge domain visualization. Proc Natl Acad Sci United States America (2004) 101 Suppl 1(Suppl 1):5303–10. doi: 10.1073/pnas.0307513100 PMC387312 14724295

[17] Chen CM. CiteSpace II: Detecting and visualizing emerging trends and transient patterns in scientific literature. J Am Soc Inf Sci Technol (2006) 57(3):359–77. doi: 10.1002/asi.20317

[18] Wu H Wang Y Tong L Yan H Sun Z. Global research trends of ferroptosis: A rapidly evolving field with enormous potential. Front Cell Dev Biol (2021) 9:646311. doi: 10.3389/fcell.2021.646311 33996807 PMC8116802

[19] Wu H Zhou Y Xu L Tong L Wang Y Liu B. Mapping knowledge structure and research frontiers of ultrasound-induced blood-brain barrier opening: A scientometric study. Front Neurosci (2021) 15:706105. doi: 10.3389/fnins.2021.706105 34335175 PMC8316975

[20] Synnestvedt MB Chen C Holmes JH. CiteSpace II: visualization and knowledge discovery in bibliographic databases. AMIA. Annual symposium proceedings. AMIA Symposium (2005) 2005:724–8. PMC1560567 16779135

[21] Ma D Guan B Song L Liu Q Fan Y Zhao L. A bibliometric analysis of exosomes in cardiovascular diseases from 2001 to 2021. Front Cardiovasc Med (2021) 8:734514. doi: 10.3389/fcvm.2021.734514 34513962 PMC8424118

[22] Yuan G Shi J Jia Q Shi S Zhu X Zhou Y. Cardiac rehabilitation: A bibliometric review from 2001 to 2020. Front Cardiovasc Med (2021) 8:672913. doi: 10.3389/fcvm.2021.672913 34136548 PMC8200471

[23] Xiao F Li C Sun J Zhang L. Knowledge domain and emerging trends in organic photovoltaic technology: A scientometric review based on CiteSpace analysis. Front Chem (2017) 5:67. doi: 10.3389/fchem.2017.00067 28966923 PMC5605557

[24] Ma D Yang B Guan B Song L Liu Q Fan Y. A bibliometric analysis of pyroptosis from 2001 to 2021. Front Immunol (2021) 12:731933. doi: 10.3389/fimmu.2021.731933 34484243 PMC8416445

[25] Chen C Hu Z Liu S Tseng H. Emerging trends in regenerative medicine: a scientometric analysis in CiteSpace. Expert Opin Biol Ther (2012) 12(5):593–608. doi: 10.1517/14712598.2012.674507 22443895

[26] Wang ZY Ma DB Pang R Xie F Zhang JX Sun DQ. Research progress and development trend of social media big data (SMBD): Knowledge mapping analysis based on CiteSpace. ISPRS Int J Geo-Inf. (2020) 9(11):16. doi: 10.3390/ijgi9110632

[27] Hirsch JE. An index to quantify an individual's scientific research output. Proc Natl Acad Sci United States America (2005) 102(46):16569–72. doi: 10.1073/pnas.0507655102 PMC1283832 16275915

[28] Frazzoni L Arribas J Antonelli G Libanio D Ebigbo A van der Sommen F. Endoscopists' diagnostic accuracy in detecting upper gastrointestinal neoplasia in the framework of artificial intelligence studies. Endoscopy (2022) 54(4):403–11. doi: 10.1055/a-1500-3730 33951743

[29] Wu L Shang R Sharma P Zhou W Liu J Yao L. Effect of a deep learning-based system on the miss rate of gastric neoplasms during upper gastrointestinal endoscopy: a single-centre, tandem, randomised controlled trial. Lancet Gastroenterol Hepatol (2021) 6(9):700–8. doi: 10.1016/s2468-1253(21)00216-8 34297944

[30] Wu L Wang J He X Zhu Y Jiang X Chen Y. Deep learning system compared with expert endoscopists in predicting early gastric cancer and its invasion depth and differentiation status (with videos). Gastrointestinal Endoscopy (2022) 95(1):92–104.e3. doi: 10.1016/j.gie.2021.06.033 34245752

[31] Wu L Xu M Jiang X He X Zhang H Ai Y. Real-time artificial intelligence for detecting focal lesions and diagnosing neoplasms of the stomach by white-light endoscopy (with videos). Gastrointestinal Endoscopy (2022) 95(2):269–280.e6. doi: 10.1016/j.gie.2021.09.017 34547254

[32] He X Wu L Dong Z Gong D Jiang X Zhang H. Real-time use of artificial intelligence for diagnosing early gastric cancer by magnifying image-enhanced endoscopy: a multicenter diagnostic study (with videos). Gastrointestinal Endoscopy (2022) 95(4):671–678.e4. doi: 10.1016/j.gie.2021.11.040 34896101

[33] Shichijo S Nomura S Aoyama K Nishikawa Y Miura M Shinagawa T. Application of convolutional neural networks in the diagnosis of helicobacter pylori infection based on endoscopic images. EBioMedicine (2017) 25:106–11. doi: 10.1016/j.ebiom.2017.10.014 PMC5704071 29056541

[34] Hirasawa T Aoyama K Tanimoto T Ishihara S Shichijo S Ozawa T. Application of artificial intelligence using a convolutional neural network for detecting gastric cancer in endoscopic images. Gastric Cancer Off J Int Gastric Cancer Assoc Japanese Gastric Cancer Assoc (2018) 21(4):653–60. doi: 10.1007/s10120-018-0793-2 29335825

[35] Niikura R Aoki T Shichijo S Yamada A Kawahara T Kato Y. Artificial intelligence versus expert endoscopists for diagnosis of gastric cancer in patients who have undergone upper gastrointestinal endoscopy. Endoscopy (2022) 54(8):780–4. doi: 10.1055/a-1660-6500 PMC9329064 34607377

[36] Wu H Li Y Tong L Wang Y Sun Z. Worldwide research tendency and hotspots on hip fracture: a 20-year bibliometric analysis. Arch osteoporosis (2021) 16(1):73. doi: 10.1007/s11657-021-00929-2 33866438

[37] Garfield E. The history and meaning of the journal impact factor. Jama (2006) 295(1):90–3. doi: 10.1001/jama.295.1.90 16391221

[38] Wu H Zhou Y Wang Y Tong L Wang F Song S. Current state and future directions of intranasal delivery route for central nervous system disorders: A scientometric and visualization analysis. Front Pharmacol (2021) 12:717192. doi: 10.3389/fphar.2021.717192 34322030 PMC8311521

[39] Li KL Chen YM Wang XQ Hu HY. Bibliometric analysis of studies on neuropathic pain associated with depression or anxiety published from 2000 to 2020. Front Hum Neurosci (2021) 15:729587. doi: 10.3389/fnhum.2021.729587 34552477 PMC8450598

[40] Kleinberg J. Bursty and hierarchical structure in streams. Data Min Knowl Discovery (2003) 7(4):373–97. doi: 10.1023/a:1024940629314

[41] Silver D Huang A Maddison CJ Guez A Sifre L van den Driessche G. Mastering the game of go with deep neural networks and tree search. Nature (2016) 529(7587):484–9. doi: 10.1038/nature16961 26819042

[42] Silver D Schrittwieser J Simonyan K Antonoglou I Huang A Guez A. Mastering the game of go without human knowledge. Nature (2017) 550(7676):354–9. doi: 10.1038/nature24270 29052630

[43] Butt NS Malik AA Shahbaz MQ. Bibliometric analysis of statistics journals indexed in web of science under emerging source citation index. SAGE Open (2021) 11(1):8. doi: 10.1177/2158244020988870

[44] Kanesaka T Lee TC Uedo N Lin KP Chen HZ Lee JY. Computer-aided diagnosis for identifying and delineating early gastric cancers in magnifying narrow-band imaging. Gastrointestinal Endoscopy (2018) 87(5):1339–44. doi: 10.1016/j.gie.2017.11.029 29225083

[45] Zhu Y Wang QC Xu MD Zhang Z Cheng J Zhong YS. Application of convolutional neural network in the diagnosis of the invasion depth of gastric cancer based on conventional endoscopy. Gastrointestinal Endoscopy (2019) 89(4):806–815.e1. doi: 10.1016/j.gie.2018.11.011 30452913

[46] Wu L Zhou W Wan X Zhang J Shen L Hu S. A deep neural network improves endoscopic detection of early gastric cancer without blind spots. Endoscopy (2019) 51(6):522–31. doi: 10.1055/a-0855-3532 30861533

[47] Horie Y Yoshio T Aoyama K Yoshimizu S Horiuchi Y Ishiyama A. Diagnostic outcomes of esophageal cancer by artificial intelligence using convolutional neural networks. Gastrointestinal Endoscopy (2019) 89(1):25–32. doi: 10.1016/j.gie.2018.07.037 30120958

[48] Luo H Xu G Li C He L Luo L Wang Z. Real-time artificial intelligence for detection of upper gastrointestinal cancer by endoscopy: a multicentre, case-control, diagnostic study. Lancet Oncol (2019) 20(12):1645–54. doi: 10.1016/s1470-2045(19)30637-0 31591062

[49] Li L Chen Y Shen Z Zhang X Sang J Ding Y. Convolutional neural network for the diagnosis of early gastric cancer based on magnifying narrow band imaging. Gastric Cancer Off J Int Gastric Cancer Assoc Japanese Gastric Cancer Assoc (2020) 23(1):126–32. doi: 10.1007/s10120-019-00992-2 PMC6942561 31332619

[50] Horiuchi Y Aoyama K Tokai Y Hirasawa T Yoshimizu S Ishiyama A. Convolutional neural network for differentiating gastric cancer from gastritis using magnified endoscopy with narrow band imaging. Digestive Dis Sci (2020) 65(5):1355–63. doi: 10.1007/s10620-019-05862-6 31584138

[51] Jiang Y Zhang Z Yuan Q Wang W Wang H Li T. Predicting peritoneal recurrence and disease-free survival from CT images in gastric cancer with multitask deep learning: a retrospective study. Lancet Digital Health (2022) 4(5):e340–50. doi: 10.1016/s2589-7500(22)00040-1 35461691

[52] Muti HS Heij LR Keller G Kohlruss M Langer R Dislich B. Development and validation of deep learning classifiers to detect Epstein-Barr virus and microsatellite instability status in gastric cancer: a retrospective multicentre cohort study. Lancet Digital Health (2021) 3(10):e654–64. doi: 10.1016/s2589-7500(21)00133-3 PMC8460994 34417147

[53] Wang X Chen Y Gao Y Zhang H Guan Z Dong Z. Predicting gastric cancer outcome from resected lymph node histopathology images using deep learning. Nat Commun (2021) 12(1):1637. doi: 10.1038/s41467-021-21674-7 33712598 PMC7954798

[54] Rasmussen SA Arnason T Huang WY. Deep learning for computer-assisted diagnosis of hereditary diffuse gastric cancer. J Pathol Trans Med (2021) 55(2):118–24. doi: 10.4132/jptm.2020.12.22 PMC7987520 33472333

