# Supplementary figures and images for: Artificial intelligence applicated in gastric cancer: A bibliometric and visual analysis via CiteSpace
Source: Front Oncol. 2023 Jan 4;12:1075974. doi: 10.3389/fonc.2022.1075974 (PMC9846739; doi:10.3389/fonc.2022.1075974)

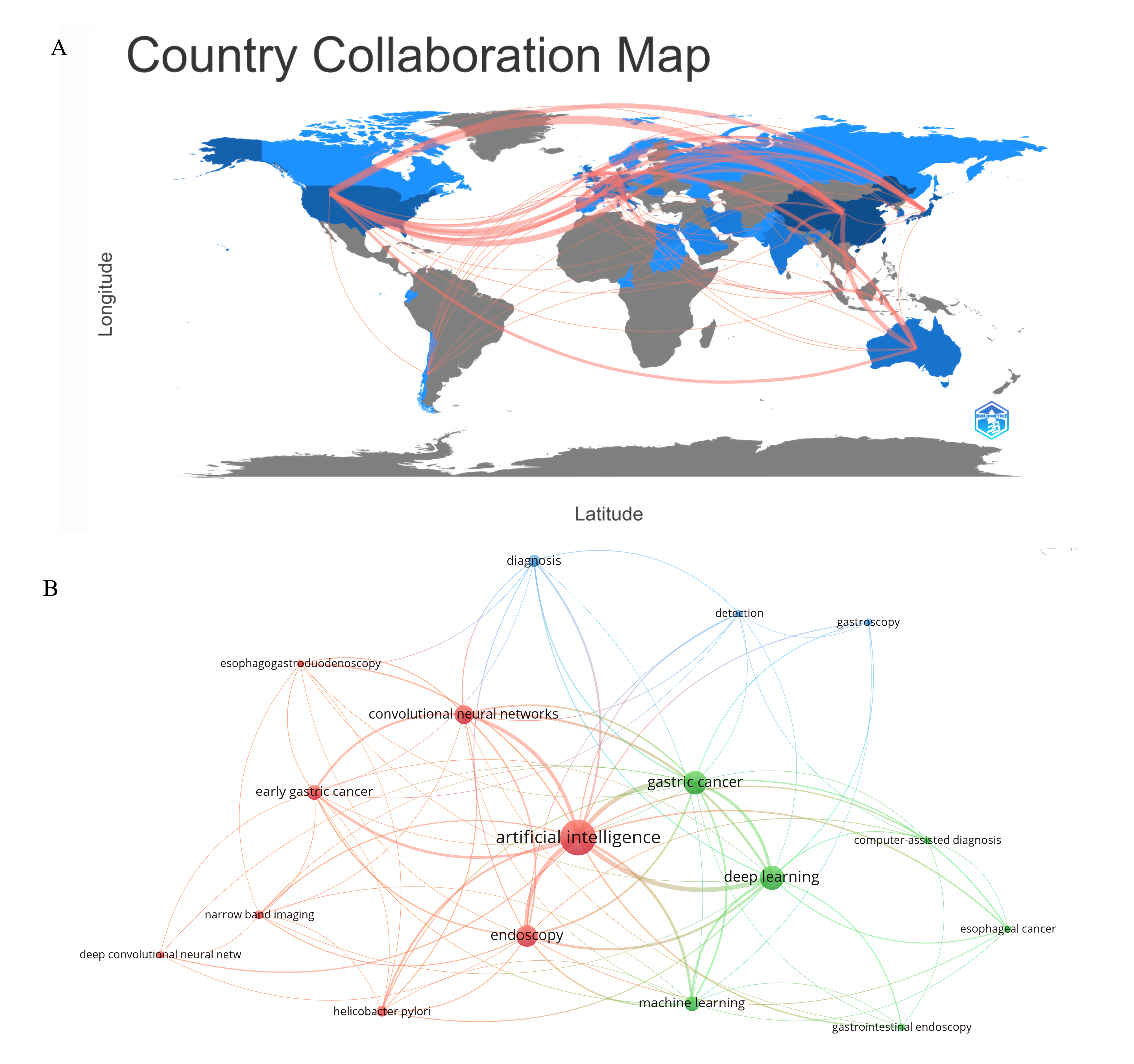

Supplement: Supplementary Figure 1 — (A) Pictorial representation of countrywide collaboration using the country collaboration map.(B) The keyword visualization map by VOSviewer. [file Image_1.tif]
